# Effects of Supervised Multimodal Exercise Interventions on Cancer-Related Fatigue: Systematic Review and Meta-Analysis of Randomized Controlled Trials

**DOI:** 10.1155/2015/328636

**Published:** 2015-06-17

**Authors:** José Francisco Meneses-Echávez, Emilio González-Jiménez, Robinson Ramírez-Vélez

**Affiliations:** ^1^Facultad de Cultura Física, Deporte y Recreación, Universidad de Santo Tomás, Carrera 9 No. 51-23, Bogotá, Colombia; ^2^Departamento de Enfermería, Facultad de Enfermería, Universidad de Granada, Campus de Melilla, Granada, Spain

## Abstract

*Objective*. Cancer-related fatigue (CRF) is the most common and devastating problem in cancer patients even after successful treatment. This study aimed to determine the effects of supervised multimodal exercise interventions on cancer-related fatigue through a systematic review and meta-analysis. *Design*. A systematic review was conducted to determine the effectiveness of multimodal exercise interventions on CRF. Databases of PubMed, CENTRAL, EMBASE, and OVID were searched between January and March 2014 to retrieve randomized controlled trials. Risk of bias was evaluated using the PEDro scale. *Results*. Nine studies (*n* = 772) were included in both systematic review and meta-analysis. Multimodal interventions including aerobic exercise, resistance training, and stretching improved CRF symptoms (SMD = −0.23; 95% CI: −0.37 to −0.09; *P* = 0.001). These effects were also significant in patients undergoing chemotherapy (*P* < 0.0001). Nonsignificant differences were found for resistance training interventions (*P* = 0.30). Slight evidence of publication bias was observed (*P* = 0.04). The studies had a low risk of bias (PEDro scale mean score of 6.4 (standard deviation (SD) ± 1.0)). *Conclusion*. Supervised multimodal exercise interventions including aerobic, resistance, and stretching exercises are effective in controlling CRF. These findings suggest that these exercise protocols should be included as a crucial part of the rehabilitation programs for cancer survivors and patients during anticancer treatments.

## 1. Introduction

The number of people diagnosed with cancer worldwide has been estimated to be as high as 10 million [1]. The respective numbers with regard to cancer survivors may reach approximately 25 million [1]. In Colombia, the National Cancer Institute (NCI) declared that malignant tumors present the third cause of mortality, increasing the mortality burden during the last sixty decades from 6% to 15% in 2002 [2, 3]. Cancer-related fatigue (CRF) is a common problem in cancer patients. Approximately, 80% to 100% of cancer patients report suffering from CRF [4]. Furthermore, it has been shown that patients continue to experience fatigue symptoms for months or years after successful treatment [4]. Several concepts of CRF have been published in the biomedical literature. The National Comprehensive Cancer Network (NCCN) [5] defined CRF as “*a distressing, persistent, subjective sense of physical, emotional and/or cognitive tiredness or exhaustion related to cancer or cancer treatment that is not proportional to recent activity and interferes with usual functioning*.*”* Besides, CRF has a severe impact on daily activities, social relationships, reintegration, and overall quality of life [6]. Some evidence has postulated that CRF may be considered as a predictor of survival for these patients [7].

Recent systematic reviews have shown that supervised exercise has the power to combat many of the side effects of cancer treatment and, thus, can be of significant benefit to patients in the short and long term [8–11]. A recent Cochrane systematic review on supervised multimodal exercise and CRF [11] concluded that exercise can be considered as a beneficial intervention for individuals with CRF and encouraged further research in this field. Benefits include improved muscle strength and body composition in patients with cancer [8, 12, 13]. The effects of multimodal exercise have been attributed to improvements in adherence and intensity [14], perhaps because of greater encouragement or confidence to work when health professional help is at hand. Therefore, it has been suggested that exercise must be individualized to specific conditions of cancer survivors to achieve the numerous benefits of exercise for the treatment of cancer, such as prevention and symptoms management [14, 15]; other authors reported that breast [16] and colon [17] cancer survivors prefer supervised exercise training over unsupervised exercise. In light of this, Lin et al. [18] compared the effects of a supervised exercise intervention with those of usual care for 12 weeks in colorectal cancer patients during chemotherapy and found significant improvements in the supervised exercise group on fatigue, physical activity level, and physical functioning, social functioning, hand-grip strength, cardiorespiratory fitness, and pain subscales of quality of life (QoL). The authors concluded that supervised exercise interventions result in larger benefits for cancer patients on these outcomes when compared with usual care. Likewise, Schneider et al. [15] reported that moderate intensity individualized exercise improves cardiopulmonary function and fatigue during and after treatment in a sample of 113 breast cancer patients. In a past review, Velthuis et al. [14] addressed a subgroup analysis of supervised exercise only, but they integrated this analysis within diagnosis groups, thus limiting the power of their conclusions. The current systematic review aims to update this growing evidence adding specific analyses regarding the effects of supervised multimodal exercise on CRF in cancer survivors.

## 2. Methods

### 2.1. Design

This systematic review is reported according to the PRISMA Statement [19]. We also followed the recommendations described in the Cochrane Handbook for Systematic Reviews of Interventions version 5.1.0 [20].

### 2.2. Literature Search

PubMed, CENTRAL, EMBASE, and OVID databases were searched between January and March 2014 independently by two blinded authors (JFM-E and RR-V). Search strategy incorporated the recommendations for a highly sensitive search strategy for the retrieval of clinical trials on PubMed [21]. The title and abstract were examined and full text was obtained if there was ambiguity regarding eligibility. The final search strategy was as follows: (randomized controlled trial) OR controlled clinical trial) OR randomized) OR trial) OR “clinical trials as topic”) AND cancer) OR neoplasm^*^) OR tumour^*^) OR tumor^*^) OR carcino^*^) OR leukaemi^*^) OR leukemi^*^) AND physical activity) OR exercise) OR resistance) OR strength) OR stretching) AND fatigue). In addition, the authors checked the reference lists of the identified studies and the meeting abstracts of the American Society of Clinical Oncology (ASCO) Annual Meeting on its website from 2004 to 2013, as well as certain journals (i.e., The Lancet Oncology, Journal of Clinical Oncology, Journal of the National Cancer Institute, Journal of Breast Cancer, The Breast Journal, and The Breast). No language restrictions were applied. Attempts were made to contact authors of trial reports if clarification was necessary. See the Appendix for further details of the search strategy procedures.

## 3. Selection Criteria

Selection criteria were built based on the PICO acronym as follows.

### 3.1. Participants

This systematic review included studies with patients (age > 18 years) diagnosed with any type of cancer regardless of the stage of diagnosis or treatment. There were no restrictions for sex, ethnicity, or race.

### 3.2. Intervention

The experimental intervention was multimodal exercise including aerobic, resistance, and stretching exercise, whilst the control intervention was conventional care, where patients did not participate in any exercise intervention program. In this sense, resistance training (RT) interventions were considered as any form of physical activity that is designed to improve muscular fitness by exercising a muscle or a muscle group against external resistance, performed in a systematic manner in terms of frequency, intensity, and duration, and is designed to maintain or enhance health-related outcomes [22]. All interventions had to be supervised by health professionals; therefore, home-based programs, telephone monitoring interventions, and cognitive approaches were excluded. Yoga and tai-chi interventions were not included because, although these interventions can be supervised by healthcare providers, they do not exert a large physiological impact (energy expenditure).

### 3.3. Outcome Measures

Primary outcome measure was CRF symptoms measured using the Functional Assessment of Cancer Therapy- (FACT-) Fatigue Scale, European Organization for Research and Treatment of Cancer Quality of Life Questionnaire (EORTC QLQ-C30), Piper Fatigue Scale (PFS), Schwartz Cancer Fatigue Scale (SCFS), and the Multidimensional Fatigue Inventory (MFI).

Selection criteria were verified independently by two blinded authors (JFM-E and RR-V) and disagreements were solved through consensus and active participation of a third author (EG-J).

### 3.4. Assessment of the Risk of Bias and Methodological Quality

The methodological quality and risk of bias of the studies were assessed using the Physiotherapy Evidence Database (PEDro) scale [23]. The PEDro scale scores the methodological quality of randomized controlled studies out of 10. Scores were based on all information available both from the published version and from communication with the authors. A score of 5 of 10 was set as the minimum score for inclusion in the current meta-analysis. Publication bias was evaluated through visual appraisal of the funnel plot built for CRF and by Egger's test (*P* < 0.05). Risk of bias was evaluated by two independent authors (JFM-E and EGJ) and a third author mediated for consensus (RR-V). Interrater reliability between authors was determined by calculating Cohen's kappa.

### 3.5. Data Extraction and Analysis

Key characteristics of the identified studies were also extracted including authors' information, publication year, study design, cancer treatment, time since diagnosis, and characteristics of the multimodal exercise interventions (mode of training, length, duration, and frequency) and effect estimates.Data extraction was conducted independently by two authors (JFM-E and RR-V) using a standard form; a third author (EG-J) arbitrated in cases of disagreement if necessary. Comprehensive Meta-Analysis Version 2.0 was used for the analyses. Continuous outcomes were pooled calculating standardized mean differences (SMD) with 95% confidence intervals (CI). Statistical heterogeneity was evaluated through visual appraisal of the forest plots and using the *I* ^2^ statistic, which was defined using the following cut-off parameters: not important heterogeneity, 0% to 40%; moderate heterogeneity, 30% to 60%; substantial heterogeneity, 50% to 90%; and considerable heterogeneity, 75% to 100% [20]. In the presence of high heterogeneity (*I* ^2^ > 50%), the pooled effects were calculated by a random effects model reported in accordance with the DerSimonian and Laird method, which considers both within-study and between-study differences [20]. On the contrary, when substantial heterogeneity was not detected, we conducted a fixed-effects model reported by using the inverse variance method [20]. We performed a metaregression analysis to explore the predictor effects of the supervised multimodal exercise characteristics, such as length (weeks), frequency (sessions per week), and duration (minutes per session), on the effect estimates. Subgroup analysis was undertaken according to the stage of anticancer treatment (active or not where reported) and by the mode of exercise training. Publication bias was evaluated with Egger's test and a funnel plot [20]. All *P* values were two-sided and were considered significant at the 0.05 level.

## 4. Results

### 4.1. Characteristics of the Included Studies

**Figure 1:**
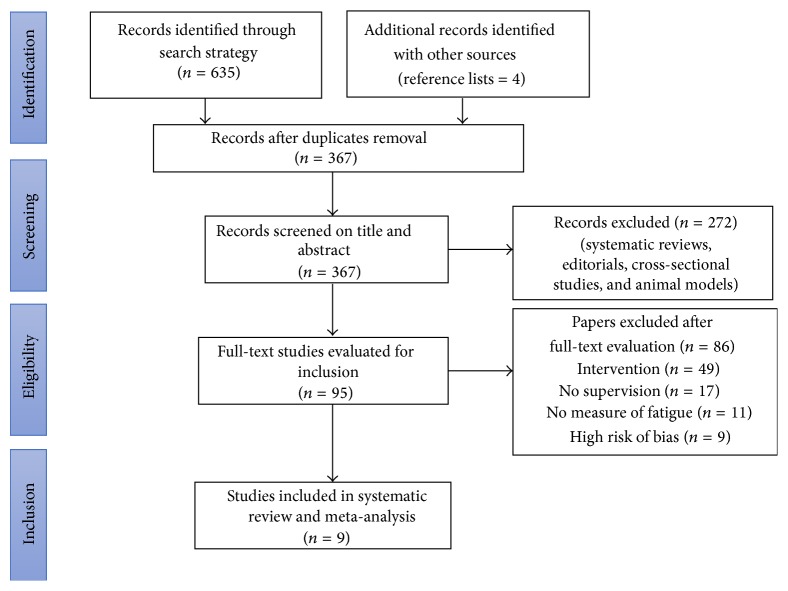


A total of nine randomized controlled trials (*n* = 772) were included [24–32] (Figure 1). The assessment of risk of bias showed a mean score of 6.4 (SD ± 1), indicating a low risk of bias and a consistent methodological quality in the studies included (Table 1). Interrater reliability between the two authors was high (mean kappa = 0.81).

CRF levels were measured using the Functional Assessment of Cancer Therapy- (FACT-) Fatigue Scale (FS) in 54.5% of the studies included, the European Organization for Research and Treatment of Cancer Quality of Life Questionnaire (EORTC QLQ-C30; 30 items) in 36.3% of the studies, the Piper Fatigue Scale (PFS) in 9.0% of the studies, and the Schwartz Cancer Fatigue Scale (SCFS) in 9.0% of the studies included.

### 4.2. Characteristics of Cancer Survivors

The mean age of the cancer survivors ranged from 46 to 60 years with a mean of 55.5 (SD ± 7.2) years. Most cancer survivors were female (*n* = 419; 54.2%). Regarding cancer treatment stage, most studies were performed during current treatment [24, 25, 28–31]; the most common treatment was chemotherapy (*n* = 522). The average number of months since cancer diagnosis was 8.2 (SD ± 10.7), although this report was not consistent across the studies included. Breast cancer was the most investigated cancer type (*n* = 6) [25–30, 32], followed by prostate cancer (*n* = 2) [28, 31], and one trial included diverse types of cancer [24]. Time since diagnosis was not consistently reported by authors, although most of the studies recruited women who were beyond five years since primary cancer diagnosis.  Table 2 summarizes the characteristics of the studies included.

### 4.3. Characteristics of Supervised Multimodal Exercise Interventions

Multimodal exercise interventions had a mean duration of 16.5 (SD ± 12.3) weeks with an average of 3 (SD ± 1.2) sessions per week. The mean session duration was 45 minutes (SD ± 29.1 min). These interventions usually started with aerobic training using stationary bicycle/cycle ergometer, walking periods followed by strengthening exercises of the upper limbs, and a final set of cooldown stretching exercises. Training intensity varied considerably among studies, ranging from 50% to 90% maximum heart rate. All studies reported preexercise screening before exercise (Table 2).

### 4.4. Follow-Up in the Studies Included

Two studies communicated follow-up for their outcome measures. Milne et al. [29] obtained data for follow-up from 97% of the participants with an adherence rate of 61.3%; the authors reported a significant increase in QoL, fatigue, anxiety, and physical fitness at 12 and 24 weeks of their follow-up period. Mutrie et al. [30] stated that the benefits in CRF observed from the exercise group continued until 6 months of follow-up.

### 4.5. Adverse Events among Studies

Two studies [26, 31] reported adverse events related to multimodal exercise interventions. Cantarero-Villanueva et al. [26] reported discomfort or low-intensity pain/stiffness in 3 patients; however, these patients completed the multimodal exercise program. Fong et al. [33] reported three adverse events where one case resulted in hospitalization or disability, one participant referred to chest pain related to exercise with negative cardiologic results, and in the aerobic group there was a case of syncope without complications.

### 4.6. Pooled Analysis

**Figure 2:**
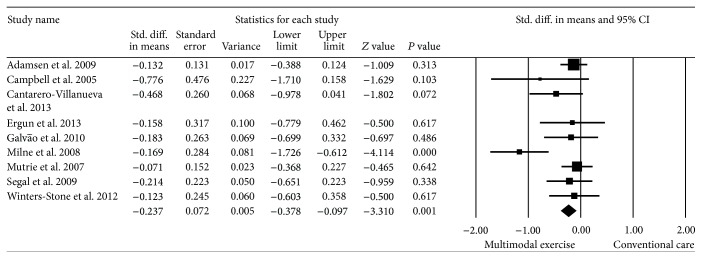
Effect estimate of supervised multimodal exercise on CRF. Standardized mean difference (SMD) was calculated for the random effects model of meta-analysis. IV, inverse of variance; CI, confidence interval.

Nine studies reported appropriate statistical measures for the meta-analysis [24–32]. Supervised multimodal exercise interventions resulted in an overall reduction in fatigue in cancer survivors (SMD = −0.23; 95% CI −0.37 to −0.09), *P* = 0.001 with low statistical heterogeneity (*I* ^2^ = 46.7%) (Figure 2).

### 4.7. Publication Bias

**Figure 3:**
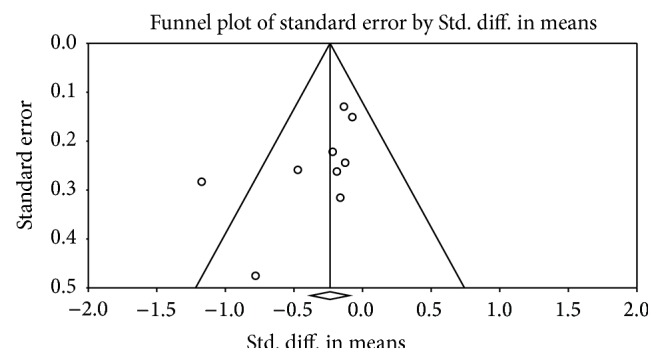
Funnel plot for the assessment of publication bias.

Visual appraisal of the funnel plot showed slight evidence of publication bias, although the reduced number of studies included could limit this analysis (Figure 3) confirmed by Egger's test (*P* = 0.04).

### 4.8. Subgroup Analyses: Stage of Treatment and Mode of Exercise

**Figure 4:**
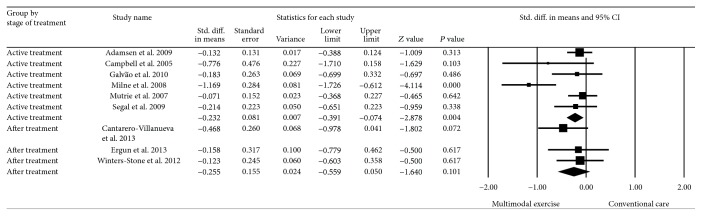
Effect estimate of supervised multimodal exercise on CRF according to anticancer treatment stage. Standardized mean difference (SMD) was calculated for the random effects model of meta-analysis. IV, inverse of variance; CI, confidence interval.

Six randomized controlled trials involved cancer patients undergoing active treatment; chemotherapy was the most common regimen [24, 25, 28–31]. The pooled effects showed overall significant improvements in CRF among cancer patients receiving anticancer treatment (SMD = −0.23, 95% CI −0.39 to −0.07), *P* < 0.0001 with moderate statistical heterogeneity (*I* ^2^ = 64%). Nonsignificant differences were found after anticancer treatment (*P* = 0.10) (Figure 4).

**Figure 5:**
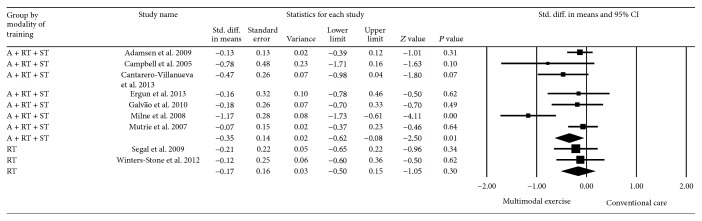
Effect estimate of supervised multimodal exercise on CRF according to the mode of exercise. A + RT + ST, aerobic exercise + resistance training + stretching; RT, resistance training. Standardized mean difference (SMD) was calculated for the random effects model of meta-analysis. IV, inverse of variance; CI, confidence interval.

With regard to mode of exercise, multimodal exercise interventions including aerobic exercise + resistance training + stretching were implemented by seven studies [24–30]; the pooled effect estimate for this subgroup showed significant reductions in CRF symptoms (SMD = −0.35, 95% CI −0.62 to −0.08), *P* = 0.01. Two studies evaluated the effects of resistance training on CRF [31, 32]. The pooled effects were not statistically significant (SMD = −0.17, 95% CI −0.50 to 0.15), *P* = 0.30 (Figure 5).

### 4.9. Metaregression: Heterogeneity and Dose-Response Relationships

**Figure 6:**
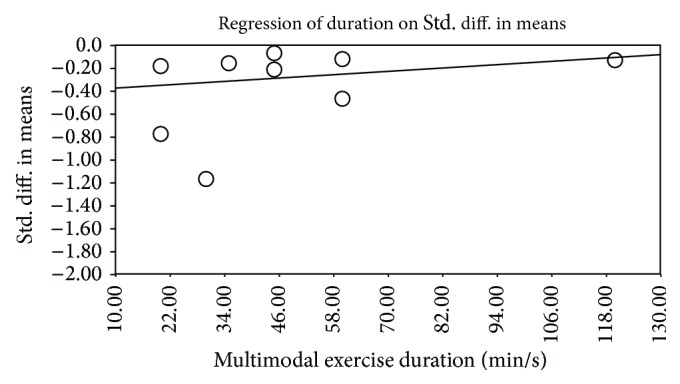
Bubble plot for the dose-response relationship between the intervention duration (minutes/session) and effect estimates changes for CRF from the nine randomized controlled trials included in the metaregression analysis (*P* = 0.04).

Our metaregression model showed that length (weeks of training), frequency (sessions/week), and duration (minutes/session) of the supervised multimodal exercise interventions were lineally associated with overall improvements in CRF levels (Tau-squared = 0.04, *P* = 0.04) (Figure 6). No significant dose-response interactions were observed for publication year and training intensity (*P* > 0.05).

## 5. Discussion

Our pooled analysis demonstrated that supervised multimodal exercise improves CRF when compared with conventional care. Similar results have been presented in prior meta-analyses on fatigue symptoms [8, 14], depression [34], and QoL [35] in cancer survivors. Further, our results are in line with those published by Fong et al. [33], where physical activity including strengthening exercises, with or without supervision, was positively associated with body composition, physical functioning, and psychological outcomes including fatigue. Nevertheless, there is insufficient information available to elucidate the physiological mechanisms for the effects of supervised multimodal exercise in reducing fatigue during cancer therapy and further research is warranted in this field [36–40].

Different from other systematic reviews, a novel finding of this review is that most interventions included in this meta-analysis were performed during an active treatment stage, especially chemotherapy. In this sense, recent findings published by Oechsle et al. [41] in a prospective randomized pilot trial found that structured exercise improved CRF in 48 patients receiving myeloablative chemotherapy who received supervised exercise five times a week with ergometer training and strength exercises for 20 min each during the hospitalization period. Our results and the current body of evidence demonstrate that RT and exercise interventions can provide significant effects on fatigue during cancer treatment, especially in patients receiving chemotherapy; however, further trials are needed to reinforce this evidence and encourage structured exercise interventions for cancer survivors undergoing anticancer treatment.

In this sense, the present meta-analysis revealed that supervised multimodal exercise leads to a significant reduction in fatigue scores in cancer survivors during and after cancer treatment. The effects of RT were not addressed by the American Cancer Society [3] that recommended RT but have been recently examined in patients undergoing cancer treatment [42]. Nevertheless, further studies are needed before RT can be recommended for cancer patients undergoing cancer therapy. Specifically, more information is required regarding the effects of initial chemotherapy and radiation therapy on muscle satellite (progenitor) cells that proliferate in response to supervised multimodal exercise [8, 42]. Clinically, this may allow the maintenance or an increase in functional performance, as well as a reduction of the risk of developing CRF, and improve perceived energy, mental capacity, and psychological status. It is not clear whether previously sedentary patients can or will adhere to an exercise program as proposed by ACSM and, if they cannot, whether the amount of exercise they do engage in will still be of benefit in terms of symptom relief (i.e., anxiety, depression, lack of sleep, and mood change) and reduction of the risk of adverse events [43].

In light of this evidence and considering that supervised exercise is broadly accepted as a beneficial intervention for cancer survivors, it is necessary to carefully conduct prescreening procedures for cancer survivors in order to achieve an adequate prescription of exercise programs, adjusting patient's specific variables, such as physiological responses and physical disturbances underlying carcinogenic process and its treatment [38]. Thus, healthcare providers who have knowledge of exercise prescription in cancer patients are ideally placed to pursue further research in this area and to prescribe physical exercise among cancer survivors [43–46]. Our findings indicate that health professionals must recognize the important benefits of adjuvant interventions in cancer survivors, such as exercise, that counteract the negative side effects of cancer treatments.

Naturally, our study has some limitations that need to be addressed. First, the average score of the quality of the studies included in this is greater than the average score for trials in physiotherapy. The risk of bias was evaluated by one author and this could be a limitation for this process, although this limitation could be counteracted considering that PEDro scale is a broadly validated tool. A second limitation is that considerable statistical heterogeneity was present in all effect estimates. Possible explanations for this heterogeneity are the diversity of sample sizes, the characteristics of strengthening programs (i.e., length, duration, and intensity) evaluated in the studies included, and the wide variety in outcome measurement tools used among studies. This heterogeneity can be observed in the forest plots (Figures 2–4).

## 6. Conclusion

In summary, the findings of this systematic review can be used to promote professional supervision in cancer rehabilitation settings and, eventually, to reinforce the conception that supervised exercise is safe and beneficial for cancer survivors through a major recommendation of strengthening programs by health professionals. A broader recommendation of exercise will lead to achievement of consistent weekly volumes of exercise if possible, including exercise twice weekly, and stretching exercises on days of nonexercise. Likewise, these results supported the implementation of personalized supervision in research, since it can optimize patient's adherence to and compliance with interventions.

## Figures and Tables

**Table 1:** Assessment of the risk of bias and methodological quality of the studies included using the PEDro scale.

Study	Random allocation	Concealed allocation	Groups similar at baseline	Participant blinding	Therapist blinding	Assessor blinding	<15% dropouts	Intention to treat analysis	Between-group difference reported	Point estimate and variability reported	Total (0 to 10)
Adamsen et al. 2009 [24]	Y	Y	Y	N	N	N	Y	Y	Y	Y	7
Campbell et al. 2005 [25]	Y	N	Y	N	N	N	Y	N	Y	Y	5
Cantarero-Villanueva et al. 2013 [26]	Y	Y	Y	N	N	Y	Y	N	Y	Y	7
Ergun et al. 2013 [27]	Y	N	Y	N	N	Y	Y	N	Y	Y	6
Galvão et al. 2010 [28]	Y	Y	Y	N	N	N	Y	Y	Y	Y	7
Milne et al. 2008 [29]	Y	Y	Y	N	N	N	Y	Y	Y	Y	7
Mutrie et al. 2007 [30]	Y	Y	Y	N	N	Y	Y	Y	Y	Y	8
Segal et al. 2009 [31]	Y	Y	Y	N	N	N	Y	Y	Y	Y	7
Winters-Stone et al. 2012 [32]	Y	Y	Y	N	N	Y	N	Y	Y	Y	7

N: no, Y: yes, and PEDro: Physiotherapy Evidence Database.

**Table 2:** Characteristics of the included studies in systematic review (*n* = 9).

Study	Design	Type of cancer	Participants^*∗*^	Intervention^*∗∗*^	Outcome measures
Adamsen et al. 2009 [24]	RCT	Mixed types of cancer	Characteristics of cancer treatment = chemotherapy *N* = 235 Female = 171 Male = 64 Exp (*n* = 118) Age (yr) = 47.2 (10.7) Con (*n* = 117) Age (yr) = 47.2 (10.6)	Exp = aerobic exercise, resistance training, and stretching Length = 6 weeks Duration = 120 min/session Frequency = 5 sessions/week Intensity = 85%–95% Con = conventional care	EORTC QLQ-C30, MOS SF-36, Leisure Time Physical Activity Questionnaire, muscular strength (1RM)

Campbell et al. 2005 [25]	RCT	Early stage (I-II) breast cancer	Characteristics of cancer treatment = chemotherapy, radiotherapy, and combination *N* = 22 Female = 22 Exp (*n* = 12) Age (yr) = 48 (10) Con (*n* = 10) Age (yr) = 47 (5)	Exp = aerobic exercise and resistance training Length = 12 weeks Duration = 20 min/session Frequency = 2 sessions/week Intensity = 60%–75% Con = conventional care	FACT-G, FACT-B, SWLS, PFS, SPAQ, 12-minute walk test

Cantarero-Villanueva et al. 2013 [26]	RCT	Breast cancer (stages I–IIIA)	Characteristics of cancer treatment = chemotherapy, radiotherapy, and combination *N* = 61 Female = 61 Exp (*n* = 32) Age (yr) = 49 (7) Con (*n* = 29) Age (yr) = 47 (8)	Exp = aerobic exercise and resistance training Length = 8 weeks Duration = 60 min/session Frequency = 3 sessions/week Intensity = 60%–75% Con = conventional care	PFS, the Spanish version of the Profile of Mood States, the “multiple sit-to-stand test,” the trunk curl static endurance test

Ergun et al. 2013 [27]	RCT	Breast cancer (stages I–IIIA)	Characteristics of cancer treatment = chemotherapy, radiotherapy, mastectomy, axillary dissection, and sentinel lymph node biopsy *N* = 60 Female = 60 Exp (*n* = 20) Age (yr) = 49.65 (8.25) Home-based exercise (*n* = 20) Age (yr) = 55.05 (6.85) Education group (*n* = 20) Age (yr) = 55.30 (10.37)	Exp = aerobic exercise and resistance training Length = 12 weeks Duration = 45 min/session Frequency = 3 sessions/week Intensity = 60%–80% Con = home-based exercise (brisk walking for 30 min/day for 3 days/week) + education programme Education group = patient information booklet that also included lymphedema-specific exercises	EORTC QLQ-C30, BFI, BDI, ELISA kit, RayBio Human Cytokine Antibody Array 3

Galvão et al. 2010 [28]	RCT	Prostate localized (93.1%) Nodal metastases (6.9%)	Characteristics of cancer treatment = chemotherapy—radiotherapy *N* = 57 Male = 57 Exp (*n* = 29) Age (yr) = 53.5 (8.7) Con (*n* = 28) Age (yr) = 52.1 (11.8)	Exp = aerobic exercise, resistance training, and stretching Length = 12 weeks Duration = 20 min/session Frequency = 2 sessions/week Intensity = 65%–80% Con = conventional care	EORTC QLQ-C30, MOS SF-36, DXA, 1 RM

Milne et al. 2008 [29]	RCT	Early stage breast cancer	Characteristics of cancer treatment = chemotherapy—radiotherapy *N* = 58 Female = 58 Exp (*n* = 29) Age (yr) = 55.2 (8.4) Con (*n* = 29) Age (yr) = 55.1 (8.0)	Exp = aerobic exercise, resistance training, and stretching Length = 12 weeks Duration = 30 min/session Frequency = 3 sessions/week Intensity = about 75% Con = delayed exercise group (DEG) completed the exercise program from 13 to 24 weeks	FACT-B, SCFS, rPARQ, Aerobic Power Index

Mutrie et al. 2007 [30]	RCT	Early stage breast cancer	Characteristics of cancer treatment = chemotherapy—radiotherapy and combination *N* = 174 Female = 174 Exp (*n* = 82) Age (yr) = 51.3 (10.3) Con (*n* = 92) Age (yr) = 51.8 (8.7)	Exp = aerobic exercise and resistance training Length = 12 weeks Duration = 45 min/session Frequency = 2 sessions/week Intensity = 50%–75% Con = conventional care	FACT-G, FACT-B, FACT-F, BDI, PANAS, SPAQ leisure time, BMI, 12-minute walk test

Segal et al. 2009 [31]	RCT	Stages I–IV prostate cancer	Characteristics of cancer treatment = radiotherapy *N* = 121 Male = 121 Exp (*n* = 40) Age (yr) = 66.2 (6.8) Resistance (*n* = 40) Age (yr) = 66.4 (7.6) Con (*n* = 41) Age (yr) = 65.3 (7.6)	Exp = aerobic exercise, resistance training, and stretching Length = 24 weeks Duration = 45 min/session Frequency = 3 sessions/week Intensity = 70%–75% Resistance = supervised resistance training of 3 times/week for 24 weeks and 2 times 8 to 12 reps of 10 exercises at 60% to 70% estimated 1 RM Con = conventional care	FACT-G, FACT-P, FACT-F, VO(2)max, 1RM, DEXA scan (percent body fat)

Winters-Stone et al. 2012 [32]	RCT	Breast cancer (stages I–IIIA)	Characteristics of cancer treatment = chemotherapy—radiotherapy *N* = 106 Female = 106 Exp (*n* = 52) Age (yr) = 62.3 (6.7) Con (*n* = 54) Age (yr) = 62.6 (6.7)	Exp = resistance training Length = 1 year Duration = 60 min/session Frequency = 2 sessions/week Intensity = 60%–80% Con = stretching placebo program	SCFS, 1-RM, PPB, hand-grip dynamometry

BDI, Beck Depression Inventory; DXA, dual-energy X-ray absorptiometry; EORTC QLQ-C30, European Organization for Research and Treatment of Cancer Quality of Life Questionnaire; RBDI, Finnish modified version of Beck's 13-item depression scale; FACT-B, FACT-F, FACT-G, and FACT-P, Functional Assessment of Cancer Therapy- (FACT-) Breast, Fatigue, General, and Prostate; FACIT-F, Functional Assessment of Chronic Illness Therapy (FACIT) questionnaire for fatigue; MOS SF-36, Medical Outcomes Study Short Form; MFSI-SF, Multidimensional Fatigue Inventory; PARQ, Physical Activity Readiness Questionnaire; PFS, Piper Fatigue Scale; PANAS, Positive and Negative Affect Scale; SPAQ, Scottish Physical Activity Questionnaire; SCFS, Schwartz Cancer Fatigue Scale; SWLS, Satisfaction with Life Scale; WHQ, Women's Health Questionnaire.

^*^Age presented with mean and SD or range where reported.

^**^Supervised multimodal exercise interventions involved a warm-up period, aerobic training (walking, cycling ergometers, and circuits), muscle strength training, and stretching exercises followed by cooldown and relaxation periods.

## Appendix

### Full Search Strategy

*PubMed Search Strategy*

1. randomized controlled trialPublication Type
2. controlled clinical trialPublication Type
3. randomi^*^edTitle/Abstract
4. trialTitle
5. “clinical trials as topic” MeSH Major Topic
6. #1 OR #2 OR #3 OR #4 OR #5
7. cancerTitle/Abstract
8. neoplasm^*^Title/Abstract
9. (tumour^*^ or tumor^*^)Title/Abstract
10. carcino^*^Title/Abstract
11. (leukaemi^*^ or leukemi^*^)Title/Abstract
12. #7 OR #8 OR #9 OR #10 OR #11
13. Physical activityTitle/Abstract
14. ExerciseTitle/Abstract
15. AerobicTitle/Abstract
16. ResistanceTitle/Abstract
17. StrengthTitle/Abstract
18. StretchingTitle/Abstract
19. #13 OR #14 OR #15 OR #16 OR #17 OR #18 OR #19
20. fatigueTitle/Abstract
21. #6 AND #12 AND #20 AND #21.

*CENTRAL Search Strategy*. (randomized controlled trial) OR controlled clinical trial) OR randomied) OR trial) OR “clinical trials as topic”) AND cancer) OR neoplasm^*^) OR tumour^*^) OR tumor^*^) OR carcino^*^) OR leukaemi^*^) OR leukemi^*^) AND physical activity) OR exercise) OR resistance) OR strength) OR stretching) AND fatigue.

*EMBASE Search Strategy*. (randomized AND controlled AND trial) OR controlled) AND clinical AND trial) OR randomied OR trial OR “clinical trials as topic”/exp OR “clinical trials as topic”) AND (cancer/exp OR cancer)) OR neoplasm^*^ OR tumour^*^ OR tumor^*^ OR carcino^*^ OR leukaemi^*^ OR leukemi^*^) AND physical AND activity) OR exercise/exp OR exercise OR resistance OR strength/exp OR strength OR) AND (fatigue/exp OR fatigue).

*OVID Search Strategy*. (randomized and controlled and trial) OR controlled) AND clinical and trial) OR randomied or trial).mp. OR “clinical trials as topic”/exp OR “clinical trials as topic”.mp.) AND (cancer/exp or cancer.mp.)) OR neoplasm^*^.mp. OR tumour^*^.mp. OR tumor^*^.mp. OR carcino^*^.mp. OR leukaemi^*^.mp. OR leukemi^*^.mp.) AND physical.mp. AND activity.mp.) OR exercise/exp OR exercise.mp. OR aerobic.mp. OR resistance.mp. OR strength/exp OR strength.mp) AND (fatigue/exp OR fatigue.mp.) mp = title, abstract, full text, caption text.
